# Effect of standard versus patient-targeted in-patient education on patients’ anxiety about self-care after discharge from cardiovascular surgery clinics

**DOI:** 10.5830/CVJA-2014-048

**Published:** 2014

**Authors:** Tülin Yıldız, Selami Gürkan, Özcan Gür, Cüneyt Ünsal, Sonay Baltacı Göktaş, Yücel Özen

**Affiliations:** Department of Surgical Nursing, School of Health, Namık Kemal University, Tekirdağ, Turkey; Department of Surgical Nursing, School of Health, Namık Kemal University, Tekirdağ, Turkey; Department of Surgical Nursing, School of Health, Namık Kemal University, Tekirdağ, Turkey; Department of Psychiatry, Faculty of Medicine, School of Health, Namık Kemal University, Tekirdağ, Turkey; Department of Surgical Nursing, School of Nursing, Maltepe University, Istanbul, Turkey; Department of Cardiovascular Surgery, Kartal Koşuyolu Education and Research Hospital, Istanbul, Turkey

**Keywords:** cardiovascular surgery, anxiety, patient’s education, coronary artery bypass surgery

## Abstract

We compared standard and patient-targeted in-patient education in terms of their effect on patients’ anxiety. One hundred and ninety-eight patients who were hospitalised for coronary artery bypass surgery were given standard education (group 1) or individualised education (group 2) on the management of their healthcare after discharge. Patients in group 2 were assessed on the patient learning needs scale and were given education according to their individual needs. The level of anxiety was measured by the state–trait anxiety inventory. Anxiety scores were significantly lower in group 2 than group 1 after education (*p* < 0.001). While state anxiety did not change after education in group 1 (*p* = 0272), it decreased significantly in group 2 (*p* < 0.001). For cardiovascular surgery patients, patient-targeted in-patient education was more effective than standard education in decreasing anxiety levels, therefore the content of the education should be individualised according to the patient’s particular needs.

## Abstract

Coronary artery bypass surgery is the absolute treatment for severe coronary artery disease, which is the leading cause of mortality globally.^1^ The surgery relieves angina symptoms, improves quality of life, and reduces cardiac-related mortality.^2^ To achive the desired benefits of coronary artery bypass surgery, it is important to decrease the risk factors for atherosclerosis and change the lifestyle of patients. For this reason, clinical practice guidelines published by several organisations have recognised the importance of patient self-care measures, and agree that patient education is an important element in the care of patients for whom cardiac surgery is planned.^3^-^5^

To this end, pre- and postoperative in-patient and discharge education have been shown to demonstrate benefits for patients in cardiovascular surgery clinics.^6^,^7^ Comprehensive in-patient education before discharge improved health outcomes such as survival and quality of life.^8^

For coronary artery bypass surgery, the duration of hospitalisation of patients ranges from four to seven days. During the hospital stay, patients usually feel anxious about surgery and about finding solutions to likely problems they may encounter after discharge. Lack of knowledge and skills necessary for home care and for the new lifestyle after discharge may slow the healing process by causing physical and psychological stress on the patient.^9^,^10^ In-patient and discharge education aims to give patients the necessary information and to decrease their anxiety levels.

Although specific elements of information and standards of patient education have been defined,^11^ there are no guidelines or standards for the education of cardiovascular surgery patients for discharge. Cardiovascular surgery clinics usually create patient education content, which focuses on postoperative care, diet, rest and exercise, and implement it via nurses who play a key role in education of hospitalised patients.^12^ Furthermore, although the advantages of in-patient education on anxiety, depression and post-discharge health outcomes were described for cardiovascular surgery patients,^13^,^14^ the effectiveness of standardised educational tools in comparison with individualised education has not been studied.

We hypothesised that in-patient education individualised according to the patient’s needs would be more targeted and effective than standardised education for patients hospitalised for coronary artery bypass surgery. On the basis of this hypothesis, we aimed in this study to compare standard and patient-targeted in-patient education in terms of their effect on patients’ anxiety about self-care after discharge.

## Methods

One hundred and ninety-eight patients who were hospitalised in the cardiovascular surgery clinic between February and August 2013 for coronary artery bypass surgery were included in the study. The study was approved by the institutional ethics committee, and all patients gave consent to participate in the study.

Patients were randomised into two groups based on the content of education: standard education (group 1, *n* = 100) and individualised education (group 2, *n* = 100) on the management of patients’ healthcare at home after discharge. Patients in group 2 were assessed with the patient learning needs scale (PLNS) to define their perception of learning needs to manage their healthcare after discharge. These patients were given education that was specified according to their individual needs.

Education was given at the same time daily from the first day of hospitalisation until the day the patient was discharged from the clinic. The duration of hospitalisation was four to five days for all patients.

The level of anxiety of patients was measured by the state–trait anxiety inventory (STAI) before education and on discharge. The STAI scores were compared between the groups and before and after education. Additionally, the effects of sociodemographic variables on the change of anxiety scores in each group were evaluated.

The education was given by one investigator to all patients in both groups. The STAIs were given to patients by another investigator who was blinded to the patients’ study groups. Only patients’ initials (not full names) and a code for education group were marked on the STAIs; thus the data entry and analysis were blinded to the study groups.

The education and outcomes were evaluated during the patients’ hospitalisation, in which time no serious complications were recorded.

The education given to in-patients by nurses in cardiovascular surgery clinics aims to help patients to meet their home-care needs before and after surgery, to facilitate getting help from the healthcare team, to accelerate the healing process, and to ensure the transition to a normal life as soon as possible. On the basis of these aims, a standard education that was developed by Ozcan *et al.*,^15^ which includes topics on drug use, coping with pain, surgical wound care, prevention of adverse effects, diet, exercise, rest, hygiene maintenance, constipation, alcohol and tobacco use, sexual activity, mood changes, emergencies, occupations and time of control visits was given face to face to group 1 patients for about 30 to 40 minutes daily. During this time, topics were repeated as necessary, taking into account individual differences. An education booklet including details on all topics was used during the education sessions. Patients in group 2 were given education that was individualised according to their specific need, which were determined by the PLNS.

## Study questionnaires

The PLNS was developed to measure patients’ learning needs in order to manage their healthcare at home after discharge from hospital.^16^ It is completed in less than 20 minutes. It has 50 items scored from 0 to 5, and seven subscales (medication, activities of living, feelings related to condition, community and follow up, treatment and complications, enhancing quality of life, and skin care), yielding a total score of 40 to 200, with higher scores indicating more importance being placed on having information at discharge. The reliability and validity of the PLNS were shown by Bubela *et al.*,^17^ and the Turkish version was also shown to be reliable and valid.^18^

The STAI is a commonly used self-report measure of anxiety, which is a four-point Likert scale and consists of 40 questions.^19^ The STAI measures two types of anxiety: state anxiety, or anxiety about an event, and trait anxiety, or anxiety level as a personal characteristic. Higher scores are positively correlated with higher levels of anxiety. It can be used to diagnose anxiety and distinguish it from depressive syndromes, and also as an indicator of caregiver distress. Its reliability and validity were demonstrated.^19^,^20^ It is offered in 12 languages, including Turkish.

## Statistical analysis

Descriptive statistical methods (frequency, percentage, mean, standard deviation) were used to summarise data, and the Kolmogorov–Smirnov test was used to determine whether data were distributed normally or not. For the comparison of quantitative data between groups, the independent samples *t*-test and Mann–Whitney *U*-test were applied for parameters with and without normal distribution, respectively.

To compare quantitative data of more than two groups with normal distribution, Kruskal–Wallis and Mann–Whitney *U*-tests were used. For in-group comparisons the paired-sample *t*-test was used. Pearson’s correlation analysis was performed to define the correlation between quantitative variables, and the outcome was expressed as correlation coefficient (*r*) and level of significance (*p*).

The level of statistical significance was set at *p* < 0.05. Statistical analyses were performed using computer software (Statistical Package for Social Sciences, Version 19.0, SPSS Inc, Chicago, Illinois, USA).

## Results

The mean ages of the patients in group 1 (*n* = 98) and group 2 (*n* = 100) were 62.1 ± 10.2 and 59.1 ± 9.8 years, respectively, and the number of male/female patients were 73/25 and 72/28, respectively. Other socio-demographic and clinical characteristics of the study patients are summarised in Table 1.

**Table 1:** Socio-demographic characteristics of the study patients

	*Group 1 (standard education) (n = 98)*	*Group 2 (individualised education) (n = 100)*	*p-value*
Gender			
Male	73 (75)	72 (72)	0.407
Female	25 (26)	28 (28)	
Age (years)	62.1 ± 10.2	59.1 ± 9.8	*0.038*
Weight (kg)	75.6 ± 11.2	80.3 ± 13.5	*0.008*
Marital status			
Married	92 (94)	94 (94)	0.602
Single	6 (6)	6 (6)	
Having children			
Yes	96 (98)	94 (94)	0.146
No	2 (2)	6 (6)	
Education			
Primary school	78 (80)	68 (68)	*0.025*
Middle school	10 (10)	13 (13)	
High school	7 (7)	6 (6)	
University	3 (3)	3 (3)	
Illiterate	0 (0)	10 (10)	
Working status			
Working	30 (31)	30 (30)	0.524
Not working	68 (69)	70 (70)	
Income ($*/TL^#^ per month)	624.0 ± 418.6	406.7 ± 202.3	*< 0.001*
Smoking			
Yes	27 (28)	31 (31)	0.353
No	71 (72)	69 (69)	
Alcohol consumption			
Yes	12 (12)	18 (18)	0.176
No	86 (88)	82 (82)	
Exercise			
Yes	18 (18)	48 (48)	< 0.001
No	80 (82)	52 (52)	
Frequency of exercise			
None	56 (57)	52 (52)	0.002
3–4 times/week	12 (12)	9 (9)	
Daily	10 (10)	30 (30)	
1–2 times/week	20 (20)	9 (9)	
On a diet			
Yes	24 (25)	36 (36)	0.054
No	74 (76)	64 (64)	

Data are given as *n* (%) or mean ± standard deviation. *$, US Dollar; ^#^TL, Turkish Lira (the exchange rate was 1 TL = $1.9961).

The study groups were homogeneous in terms of gender, marital status, having children, working status, smoking, alcohol consumption, and being on a diet (*p* > 0.05). There was no significant difference between group 1 and group 2 in terms of age, weight, education, income and frequency of exercise (*p* < 0.05) (Table 1).

The STAI scores showed no statistical difference between the study groups before education (*p* = 0.168 and *p* = 0.583, respectively). However, both anxiety scores were significantly lower in group 2 than in group 1 after education (*p* < 0.001 for STAI scores). Furthermore, while state anxiety did not change in group 1 after education (*p* = 0272), it decreased significantly in group 2 (*p* < 0.001). On the other hand, trait anxiety decreased significantly with education in both groups (*p* < 0.001 for both groups) (Tables 2, 3).

**Table 2:** State anxiety scores from the STAI

	*Group 1 (standard education) (n = 98)*	*Group 2 (individualised education) (n = 100)*	*t-value*	*p-value*
Before education	54.34 ± 5.06	55.23 ± 3.94	1.38	0.168
After education	54.96 ± 4.47	26.93 ± 2.56	–54.01	< 0.001
*t*-value	0.50	65.77		
*p*-value	0.275	< 0.001		

**Table 3:** Trait anxiety scores from the STAI

	*Group 1 (standard education) (n = 98)*	*Group 2 (individualised education) (n = 100)*	*t-value*	*p-value*
Before education	47.36 ± 6.71	46.91 ± 4.48	–0.55	0.583
After education	43.41 ± 5.79	34.45 ± 4.83	–11.82	< 0.001
*t*-value	4.71	33.83		
*p*-value	< 0.001	< 0.001		

Socio-demographic variables had a limited effect on the change in STAI scores with education in both study groups (Table 4). In the standard-education group (group 1), none of the socio-demographic variables had an effect on the education-induced change in state and trait anxiety scores, except gender; in male patients, a larger change in trait anxiety was found with education (*p* = 0.017).

**Table 4:** Effect of socio-demographic variables on the change in STAI anxiety scores with education

	*Group 1 (standard education, n = 98)*				*Group 2 (individualised education, n = 100)*			
	*Change in state anxiety with education*	*p-value*	*Change in trait anxiety with education*	*p-value*	*Change in state anxiety with education*	*p-value*	*Change in trait anxiety with education*	*p-value*
Gender								
Male	–0.58 ± 5.51	0.922	2.80 ± 7.78	0.017	28.42 ± 4.50	0.513	12.33 ± 3.72	0.410
Female	–0.76 ± 6.02		7.32 ± 8.99		28.00 ± 3.80		12.79 ± 3.64	
Marital status								
Married	–0.47 ± 5.51	0.265	3.78 ± 8.02	0.750	28.44 ± 4.21	0.427	12.67 ± 3.65	0.017
Single	–3.00 ± 7.18		6.50 ± 12.49		26.17 ± 5.57		9.17 ± 2.56	
Having children								
Yes	–0.72 ± 5.59	0.314	3.88 ± 8.06	0.860	28.13 ± 4.30	0.101	12.45 ± 3.71	0.821
No	4.00 ± 7.07		7.50 ± 21.92		31.00 ± 3.69		12.67 ± 3.56	
Education								
Primary school	–0.50 ± 5.80	0.965	4.49 ± 7.85	0.403	28.10 ± 4.13	0.895	12.00 ± 3.67	0.131
Middle school	–0.90 ± 2.77		5.40 ± 6.92		29.54 ± 5.08		14.31 ± 4.55	
High school	–1.00 ± 6.86		–1.00 ± 11.26		29.50 ± 5.72		12.67±2.16	
University	–2.00 ± 7.21		–3.33 ± 13.65		27.00 ± 3.61		14.00 ± 1.00	
Illiterate					27.70 ± 4.08		12.60 ± 3.37	
Working status								
Working	–2.27 ± 4.86	0.056	2.07 ± 9.27	0.331	29.77 ± 4.03	0.029	12.93 ± 3.50	0.339
Not working	0.10 ± 5.80		4.78 ± 7.76		27.67 ± 4.29		12.26 ± 3.76	
Smoking								
Yes	–0.56 ± 4.73	0.936	4.19 ± 9.10	0.790	28.74 ± 4.09	0.555	13.03 ± 4.07	0.248
No	–0.65 ± 5.95		3.86 ± 8.04		28.10 ± 4.41		12.20 ± 3.50	
Alcohol consumption								
Yes	–1.08 ± 3.83	0.761	2.92 ± 8.95	0.774	28.56 ± 3.99	0.850	12.33 ± 3.53	0.850
No	–0.56 ± 5.83		4.09 ± 8.25		28.24 ± 4.39		12.49 ± 3.74	
Exercise								
Yes	–2.00 ± 5.96	0.150	2.17 ± 7.82	0.142	28.50 ± 4.32	0.618	13.29 ± 3.97	0.048
No	–0.31 ± 5.53		4.35 ± 8.40		28.12 ± 4.32		11.69 ± 3.25	
Frequency of exercise								
None	–0.23 ± 5.07	0.552	3.95 ± 7.78	0.996	28.12 ± 4.32	0.393		
3–4 times/week	–0.50 ± 5.25		4.42 ± 8.59		28.22 ± 3.35			
Daily	–3.00 ± 7.94		2.60 ± 11.09		27.83 ± 4.36			
1–2 times/week	–0.60 ± 6.10		4.35 ± 8.61		31.00 ± 4.58			
On a diet								
Yes	–0.50 ± 5.36	0.944	3.33 ± 6.88	0.459	28.67 ± 4.42	0.650	13.25 ± 4.02	0.172
No	–0.66 ± 5.73		4.15 ± 8.74		28.09 ± 4.26		12.02 ± 3.43	

In the individualised-education group (group 2), only working status had a significant effect on the education-induced change in state anxiety (*p* = 0.029). Marital status (*p* = 0.017) and exercise (*p* = 0.048) had significant effects on the education-induced change in trait anxiety.

In both study groups, there was no significant correlation between education-induced change in state or trait anxiety scores and age, weight or income of patients, except a slight significant negative correlation between change in trait anxiety score and weight of patients in group 1 (*r* = –0.257; *p* = 0.011). Accordingly, as weight increased, the education-induced reduction of trait anxiety decreased in patients receiving standard education (Table 5).

**Table 5:** Correlation (*r*, correlation coefficient) between change in anxiety score from the STAI and age, weight and income level

		*Group 1 (standard education, n = 98)*		*Group 2 (individualised education, n = 100)*	
		*Change in state anxiety with education*	*Change in trait anxiety with education*	*Change in state anxiety with education*	*Change in trait anxiety with education*
Age (years)	r	–0.072	–0.073	–0.172	–0.020
	p	0.479	0.473	0.088	0.845
Weight (kg)	r	0.024	–0.257	–0.133	–0.057
	p	0.818	0.011	0.188	0.575
Income	r	–0.093	0.127	–0.051	0.091
	p	0.360	0.211	0.611	0.370

## Discussion

In this prospective, hospital-based, blind-analysis study, we found that in-patient education was effective in decreasing anxiety levels of patients who were hospitalised in a cardiovascular clinic for coronary artery bypass surgery. More remarkably, our findings showed that in-patient education targeted to the patient’s particular needs provided more benefit than standard education in decreasing anxiety of patients about self-care after discharge.

Patients hospitalised for coronary artery bypass surgery in cardiovascular surgery clinics are usually under psychological pressure about the surgery and their new life after discharge. This pressure is greater if they are not aware of and ready for the problems that may develop during home care after discharge. Studies have shown that patients for whom cardiac surgery is planned, want to know about their disease and its treatment, complications and measures that should be taken, and lifestyle after surgery.^9^ Goodman^10^ evaluated what information and support patients feel they need in the six-week rehabilitation period following discharge after cardiac surgery, and pointed to the need for improvements in the psychological preparation of patients for discharge after cardiac surgery.

In this study, therefore, we focused on the effect of in-patient education on patients’ anxiety levels. We used the STAI, a well-established anxiety tool, to determine their anxiety about the period after discharge (presented as state anxiety) and general level of anxiety (presented as trait anxiety). Our study population had high levels of both state and trait anxiety on the first day of hospitalisation before in-patient education.

It has been established in many studies that in-patient and discharge education is essential and beneficial to decrease anxiety levels and depression, hospital re-admissions, non-adherence to medication, and to improve quality of life, survival, and to enable patients to retain a sense of control in their lives.^7^,^8^,^13^ Education at the time of hospital discharge provides improved clinical outcomes, increased adherence to self-care measures, and reduced cost of care in patients with cardiac disease.^6^

Therefore, patient education by nurses during the hospitalisation period is suggested in the common guidelines as part of the routine pharmacological and surgical treatment of patients with cardiovascular diseases.^12^ The Joint Commission on Accreditation of Healthcare Organisation defined the standards of patient education by nurses.^11^ However, there are currently no guidelines or standards on how to educate cardiovascular surgery patients for discharge with regard to the amount or content of the information necessary to be effective. Therefore we aimed to compare the effectiveness of standard and patient-targeted in-patient education in decreasing patients’ anxiety levels in the cardiovascular clinic.

To measure patients’ learning needs to manage their healthcare at home after discharge from hospital, we assessed them using the PLNS.^17^,^18^ We modified the standard education for each individual patient according to the results of the PLNS, and applied this modified and individualised education to the patients in group 2.

We showed that state anxiety decreased significantly only after patient-targeted education but not after standard education given during the hospitalisation period. On the other hand, trait anxiety, which represents the personal anxiety level of the subject independent of the event, decreased in both groups, being significantly lower on discharge in the individualised-education group than in the standard-education group. Therefore, patient-individualised education was more effective than standard education in decreasing both state and trait anxiety levels in cardiovascular surgery patients.

Additionally, we evaluated the effect of socio-demographic factors, and found that these variables had a limited effect on the change in STAI scores. This suggests that the change in anxiety was mainly attributable to the effect of in-patient education.

Our findings are in line with previous studies reporting the advantage and necessity of in-patient education for patients hospitalised for cardiovascular surgery,^7^,^8^,^13^ but we are the first to demonstrate the superiority of patient-targeted education above standard education in decreasing anxiety levels of patients. Since present evidence in the literature suggests that the psychological stability of patients is associated with better physiological parameters after the procedure and early surgical recovery,^21^-^23^ we propose that decreased anxiety provided by patient-targeted education is related to better clinical outcomes after cardiovascular surgery. However, the effect of decreased anxiety levels produced by patient-targeted education on morbidity and mortality rates after cardiovascular surgery should be evaluated in further clinical studies.

Individual instruction of patients is important to accelerate healing after surgery and to enable the patient’s return to social and business life in the shortest possible time. In this context, although gender, marital status, having children, working status, smoking, alcohol consumption, being on diet, and hospitalisation duration showed similarities between the study groups, decreased anxiety levels in patients who received patient-targeted in-patient education resulted in increased levels of selfcare and self-confidence.

The main limitations of this study are that we did not follow up on the patients after discharge, and did not evaluate the outcomes of standard versus patient-targeted in-patient education on patients’ work and health outcomes after discharge. Nevertheless, it is the first study comparing patient-targeted and standard in-patient education in a cardiovascular surgery clinic, where patients have high levels of anxiety about the period after discharge. The advantage of patient-targeted education over standard education in lowering patients’ anxiety levels may decrease patients’ physical and psychological stress levels and therefore provide better outcomes after surgery.

## Conclusion

Education of cardiovascular surgery patients during hospitalisation about subsequent home care and the new lifestyle after discharge is necessary to decrease patients’ anxiety levels, and should be implemented as part of the surgical and pharmacological treatment of patients. Since patient-targeted in-patient education was more effective than standard education in decreasing patients’ anxiety, the content of the education should be individualised according to the patient’s particular needs. In this way, in-patient education will be more beneficial in decreasing anxiety levels of patients and provide more effective use of resources. Studies on the effect of patient-targeted in-patient education on post-discharge health outcomes are needed to test the further advantages of this type of education.
